# Response to “The Reality of Pervasive Transcription”

**DOI:** 10.1371/journal.pbio.1001102

**Published:** 2011-07-12

**Authors:** Harm van Bakel, Corey Nislow, Benjamin J. Blencowe, Timothy R. Hughes

**Affiliations:** 1 Banting and Best Department of Medical Research and Terrence Donnelly Centre for Cellular and Biomolecular Research, University of Toronto, Toronto, Ontario, Canada; 2 Department of Molecular Genetics, University of Toronto, Toronto, Ontario, Canada; University of California Berkeley, United States of America

Clark et al. criticize several aspects of our study [1], and specifically challenge our assertion that the degree of pervasive transcription has previously been overstated. We disagree with much of their reasoning and their interpretation of our work. For example, many of our conclusions are based on overall sequence read distributions, while Clark et al. focus on transcript units and seqfrags (sets of overlapping reads). A key point is that one can derive a robust estimate of the relative amounts of different transcript types without having a complete reconstruction of every single transcript.

In this brief response, we first revisit what is meant by pervasive transcription, and its potential significance. We then discuss the major points raised by Clark et al. in the order presented in their critique. Finally, we demonstrate that conclusions very similar to those of our original study are reached with a dataset with far greater read depth, obtained by strand-specific sequencing of rRNA-depleted total RNA from a single cell type.

## The Meaning of “Pervasive”, and the Importance of Transcript Abundance

Clark et al. define pervasive transcription of a genome to mean “that the majority of its bases are associated with at least one primary transcript”, which is the same definition used in the ENCODE 1% paper [2]. We believe that this specific claim is not contested, nor is it particularly interesting. First, it has long been assumed that roughly half of the human genome comprises introns [3]. Second, the mechanisms that control the positions of initiation and termination of Pol II transcription, as well as RNA processing, are imperfect, such that low-level background transcripts from both physiologically relevant and non-canonical sites arise [4]–[6]. Blockage of surveillance mechanisms that normally degrade such “cryptic” transcripts greatly increases their abundance [7],[8].

We acknowledge that the phrase quoted by Clark et al. in our Author Summary should have read “stably transcribed”, or some equivalent, rather than simply “transcribed”. But this does not change the fact that we strongly disagree with the fundamental argument put forward by Clark et al., which is that the genomic area corresponding to transcripts is more important than their relative abundance. This viewpoint makes little sense to us. Given the various sources of extraneous sequence reads, both biological and laboratory-derived (see below), it is expected that with sufficient sequencing depth the entire genome would eventually be encompassed by reads. Our statement that “the genome is not as not as pervasively transcribed as previously reported” stems from the fact that our observations relate to the relative quantity of material detected.

Of course, some rare transcripts (and/or rare transcription) are functional, and low-level transcription may also provide a pool of material for evolutionary tinkering. But given that known mechanisms—in particular, imperfections in termination (see below)—can explain the presence of low-level random (and many non-random) transcripts, we believe the burden of proof is to show that such transcripts are indeed functional, rather than to disprove their putative functionality.

## Contradiction of Previous Reports

The fact that our analyses contradict previous reports is precisely why we emphasized the lack of abundant pervasive transcription in our study. Clark et al. cite papers that have previously documented pervasive transcription, and point out that several different approaches have been used as confirmation. We believe that Clark et al. misinterpret what can be claimed from much of the literature in this area, and fail to acknowledge known weaknesses in some of these studies. We previously reviewed these issues [9]. For example, the number of transfrags detected in permuted tiling array data can be as high as it is in the real data [10]. In addition, a common form of “validation” in these papers is RT-PCR or RACE, but these approaches are generally semi-quantitative at best and are prone to artefacts such as template switching, which readily produces chimeric transcripts in vitro ([11] and references therein). Indeed, we note that in the ENCODE 1% study [2] repeatedly cited by Clark et al., 75 of the 100 negative controls (randomly selected non-transfrag regions) were actually detected by RACE, making the “validation” rate for negative controls only slightly lower than that for the intronic and intergenic transfrags (86%–88%). Thus, either the tiling arrays or RACE assays are highly error prone. The contention of Clark et al. that “any estimate of the pervasiveness of transcription requires inclusion of all data sources” is flawed, because if one introduces erroneous data from even a single source, the estimate becomes worse.

## Accuracy of Tiling Arrays

We agree that results obtained from tiling arrays should improve with increased tiling probe coverage. Nonetheless, the study by Agarwal et al. [12], which is highlighted by Clarke et al., shows that RNA-Seq is more accurate than tiling arrays, even when using arrays with the 5-nt resolution that Clarke et al. emphasize is important. Agarwal et al. also found that “about 4 million reads are required to match the sensitivity of two tiling array replicates”, which is counter to the arguments Clarke et al. raise regarding sampling artefacts in RNA-Seq (also see below). Even the precision recall curves shown for the new data generated by Clark et al. display a higher AUC for RNA-Seq than for tiling arrays. We believe that previous conclusions based on tiling array data that are not confirmed by RNA-Seq should be revisited.

## Depth of Sequencing, Analysis of Poly-A RNA, Dismissal of Introns, and Lack of Strand Specificity

Our previous paper acknowledged these caveats [1], and we included an analysis of previously published rRNA-depleted samples [13], which seems to have been ignored by Clark et al. It is important to note that assessment of the relative abundance of different transcript types would not be greatly affected by the depth of sequencing; it is the detection of very rare transcripts that is compromised.

Most of these concerns can be further addressed with additional data, and we present such an example here. We used strand-specific SOLiD sequencing to analyze rRNA-depleted RNA from a homogeneous cell line (293T cells), obtaining ∼131 million uniquely mapping 50-base reads. A conventional estimate is that 1 RPKM (reads per kb per million reads) of mRNA represents approximately one transcript copy per cell in human cells [14]. If we liberally estimate that there could be twice as much “dark matter” as there is mRNA, then 0.5 RPKM would be approximately equivalent to one copy per cell (rRNA, tRNA, snRNA, and snoRNA were removed from this analysis). Thus, for a 1-kb transcript present at one copy per cell, we expect ∼65.5 reads; for one copy per 10 cells, we expect ∼6.55 reads, etc.

**Figure 1:**
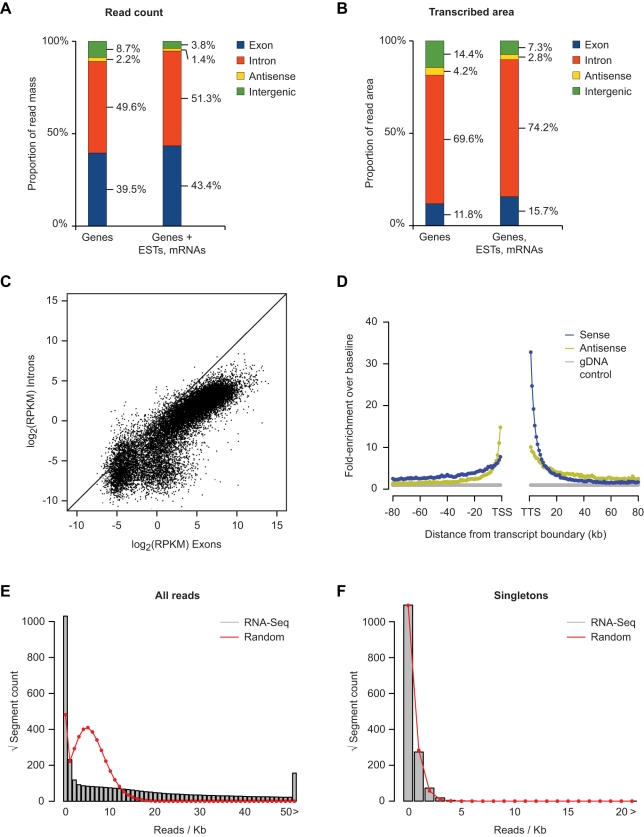
rRNA-depleted RNA-Seq analysis of HEK-293T cells. (A) Proportion of reads with a unique match to known genes (left), or known genes supplemented with mRNAs and spliced ESTs (right). Reads were sequentially matched against a non-redundant set of known genes, mRNA and spliced EST data. Any remaining reads were classified as “other”. The known gene set was derived from the UCSC, NCBI, and ENSEMBL genome databases and did not include any lincRNA annotations or processed transcripts. (B) Same as in (A), but considering the total amount of transcribed genomic area. (C) Correlation between RPKB (reads per Kb) for introns and exons of known genes. (D) Relative enrichment of RNA-Seq read frequency in intergenic regions as a function of the distance to 5′ and 3′ ends of annotated genes in the human genome. The median of read frequencies in either orientation between 80 and 100 kb was used as baseline. (E) Rootograms showing the distribution of the total number of RNA-Seq reads per kb of intergenic sequence outside 10-kb gene-flanking regions, compared to the expected random distribution for the same number of reads (red line). (F) Same as (E), but considering only intergenic transcribed regions with single-read coverage (singletons).

We repeated our previous analysis pipeline on this dataset, adapting to strand specificity. We find that ∼40% of all uniquely mapping reads are from coding exons, and ∼50% are from introns (Figure 1A). Measured by area detected by at least one read, the majority of the transcribed area corresponds to introns (Figure 1B). The density of intronic reads is 9.7% that of the exons from the same gene, on average, with a strong correlation between the read count from introns and exons from the same gene over several orders of magnitude (Figure 1C). Given that a typical mRNA is present at one or a few copies per cell, this shows that we are detecting unstable processing intermediates of even rare transcripts. Only ∼2% of all reads are antisense to genes (Figure 1A), while ∼4% are intergenic (taking into consideration areas corresponding to all known genes, ESTs, and mRNAs). As we observed previously, the majority of these intergenic reads (53.6%) are found within 10 kb of a gene end. Figure 1D shows the distribution of intergenic reads relative to gene ends.

The data presented in Figure 1D suggest that incomplete termination is likely responsible for transcripts extending far beyond 10 kb, as the enrichment over baseline in the sense orientation after the TTS extends to roughly 30 kb. In addition, at distances from 10 kb to at least 80 kb from gene ends, there is a tendency for intergenic transcripts to be oriented toward the gene being assessed. This is easily explained as a result of incomplete termination from neighboring genes: if the neighboring gene is oriented towards the gene being assessed, it is more likely to produce intergenic transcripts, which will also be oriented towards the gene being assessed. Many antisense transcripts also appear to be explained by incomplete termination of neighboring genes: 54.3% of antisense reads are within 20 kb of the 3′ end of a neighboring gene.

As we reported previously, the reads per Kb for singleton (i.e., isolated) distal intergenic transcripts (>10 kb from genes) is nearly identical to a Poisson (i.e., random) distribution, while a relatively small number of loci contain dozens to hundreds of reads per Kb (Figure 1E, 1F). As Clark et al. note, assembling full transcripts from short-read data remains a challenging computational problem. Our initial assessment, however, suggests that many of these transcripts are likely to represent unannotated exons of coding genes, lincRNAs, and enhancer-derived RNAs (unpublished data).

In summary, we disagree with the fundamental assertion that it is the total area of transcribed sequence that is most important. Our published claim that most “dark matter” transcripts can be explained as by-products of the process of transcribing known genes holds: whether they are functional remains to be seen, but the notion that because they exist they are likely to be functional violates Occam's Razor. We do not dispute that many new independent intergenic transcripts may be functional, nor that new functional RNAs can reside within introns. Indeed, the discovery of new RNAs is changing our view of how the genome functions and evolves, and the original motivation of our previous study [1] was to identify and characterize novel transcripts. Nonetheless, in contrast to the conclusions of previous studies, we observe that the abundance of “dark matter” transcripts is low, in aggregate, and the number of well-supported independent RNAs is still relatively small. It is also worth noting that a recently quoted estimate for the total number of GENCODE lincRNAs is ∼12,000 [15]. This number is substantially smaller than the number of known genes and ncRNAs, and given that lincRNA genes are typically shorter than protein coding genes [16], the number of lincRNA exons is an order of magnitude less than the number of known exons of protein-coding genes—which only represent ∼2% of the genome. In our view, a compelling wealth of evidence now supports our statement that “the genome is not as pervasively transcribed as previously reported”. We believe that the results from our study will facilitate more focused efforts directed at the characterization of biologically important transcripts.
